# Scabies and Impetigo Prevalence and Risk Factors in Fiji: A National Survey

**DOI:** 10.1371/journal.pntd.0003452

**Published:** 2015-03-04

**Authors:** Lucia Romani, Josefa Koroivueta, Andrew C. Steer, Mike Kama, John M. Kaldor, Handan Wand, Mohammed Hamid, Margot J. Whitfeld

**Affiliations:** 1 Kirby Institute, University of New South Wales, Sydney, Australia; 2 Ministry for Social Welfare, Suva, Fiji; 3 Centre for International Child Health, University of Melbourne, Australia; 4 Ministry of Health, Suva, Fiji; 5 Mugda 500 Bed General Hospital, Dhaka, Bangladesh; 6 Department of Dermatology, St Vincent’s Hospital, Sydney, Australia; Queensland Institute for Medical Research, AUSTRALIA

## Abstract

**Background:**

Scabies is recognised as a major public health problem in many countries, and is responsible for significant morbidity due to secondary bacterial infection of the skin causing impetigo, abscesses and cellulitis, that can in turn lead to serious systemic complications such as septicaemia, kidney disease and, potentially, rheumatic heart disease. Despite the apparent burden of disease in many countries, there have been few large-scale surveys of scabies prevalence or risk factors. We undertook a population-based survey in Fiji of scabies and impetigo to evaluate the magnitude of the problem and inform public health strategies.

**Methodology/Principal Findings:**

A total of 75 communities, including villages and settlements in both urban and rural areas, were randomly selected from 305 communities across the four administrative divisions, and all residents in each location were invited to participate in skin examination by trained personnel. The study enrolled 10,887 participants. The prevalence of scabies was 23.6%, and when adjusted for age structure and geographic location based on census data, the estimated national prevalence was 18.5%. The prevalence was highest in children aged five to nine years (43.7%), followed by children aged less than five (36.5%), and there was also an indication of prevalence increasing again in older age. The prevalence of scabies was twice as high in iTaukei (indigenous) Fijians compared to Indo-Fijians. The prevalence of impetigo was 19.6%, with a peak in children aged five to nine years (34.2%). Scabies was very strongly associated with impetigo, with an estimated 93% population attributable risk.

**Conclusions:**

As far as we are aware, this is the first national survey of scabies and impetigo ever conducted. We found that scabies occurs at high levels across all age groups, ethnicities, and geographical locations. Improved strategies are urgently needed to achieve control of scabies and its complications in endemic communities.

## Author Summary

Recently added to the World Health Organization list of neglected tropical diseases, scabies is an under-recognised cause of morbidity in many developing countries, due to secondary bacterial infection of the skin that can in turn lead to serious systemic complications such as kidney disease and, potentially, rheumatic heart disease. Despite the apparent burden of disease in many countries, there have been few large-scale surveys of scabies prevalence or risk factors. We undertook a population-based survey in Fiji of scabies and impetigo to evaluate the magnitude of the problem and inform public health strategies. We examined 10,887 people across 75 communities in all four geographical divisions of Fiji, covering both urban and rural areas. The national prevalence of scabies and impetigo was 23.6% and 19.6% respectively, and highest in children aged 5–9 years. We found that scabies was very strongly associated with impetigo. Scabies was twice as high in iTaukei (indigenous) Fijians compared to Indo-Fijians. Our study shows that scabies occurs at high levels across all age groups, ethnicities, and geographical locations. Improved strategies are urgently needed to achieve control of scabies and its complications in endemic communities.

## Introduction

Scabies is a skin disease caused by infestation with a tiny mite (*Sarcoptes scabiei*) that burrows under the skin and is transmitted through close personal contact [1]. The direct effect of scabies is debilitating itching, leading to scratching, which is in turn followed by complications due to bacterial infection of the skin, ranging from impetigo, abscesses and cellulitis, through to septicaemia and even death [1]. Bacterial infections secondary to scabies can also lead to more serious sequelae associated with group A streptococcal infection such as rheumatic fever and glomerulonephritis [2–6].

Scabies and its complications are considered endemic in most Pacific Island countries and in many other tropical countries including in Africa largely on the basis of anecdotal reporting. Prevalence surveys of scabies have been conducted in localized areas of a limited number of countries [7–14]. These studies have generally confirmed high levels of scabies in these locations but none have been national that we are aware of, and have not been sufficiently broad-based to provide the basis for developing and informing national disease control strategies.

Scabies was recently added to the World Health Organization’s list of Neglected Tropical Diseases, but has generally not been recognised as a public health priority in most developing countries, perhaps because of the absence of large scale surveys to fully define its extent and risk factors [3]. In order to provide comprehensive information on the prevalence of scabies and impetigo and the effect of demographic risk factors in a country believed to be highly endemic for these conditions, we undertook a national prevalence survey in Fiji.

## Methods

### Setting

Fiji is an island nation located in the South Pacific region. Its population, estimated at 851,744 in 2011 is located on an archipelago of more than 300 islands, and made up predominantly of iTaukei (indigenous) Fijians (57%) and Indo-Fijians (38%) [15].

Fiji is ranked 96 out of 187 countries in the United Nations Human Development Index, with a Gross Domestic Product (GDP) per capita of US dollars 4,728 in 2012 [16,17].

Fiji’s national territory is divided into four administrative divisions and twenty medical subdivisions. Primary care is provided at the village and district level, primarily by nurses, and secondary and tertiary medical services are provided by three divisional hospitals of which two are considered as specialised national hospitals. The training component of the study was conducted at Tamavua Twomey Hospital, the national referral hospital for skin diseases, leprosy and tuberculosis, located in the capital Suva.

### Study design

A cross-sectional survey of the national population was conducted to assess the prevalence of scabies and impetigo. The study, conducted in 2007, followed the sampling methodology of a survey previously undertaken as part of the Pacific Elimination of Lymphatic Filariasis programme (PacELF) in Fiji.

### Study sampling

For the lymphatic filariasis survey, the Fiji population was divided into 23 strata designated by medical area within which there were 305 administrative units [18]. Under Fijian administrative designation, each unit was either a village or a settlement. Generally villages are rural and settlements urban or peri-urban, but there is some cross-over. The survey used a stratified cluster sampling method with stratification at the medical area level and administrative units designated as clusters. The target sample size was 17,250. After stratification 198 administrative units were randomly selected. For villages and settlements with a population above 200, a sample of 200 randomly chosen residents was invited to participate, whereas for those with less than 200 the entire population was invited.

Due to resource constraints, the scabies survey was not able to aim for a sample size as large as that of the lymphatic filariasis survey, so a member of the Fiji Ministry of Health randomly selected a subset (n = 77) of the PacELF survey sites. This number was chosen so that a minimum total sample size could be achieved within each of the four divisions. In contrast to the PacELF survey, all members of selected communities were invited to participate (rather than limiting to 200 in communities with populations above 200). Of the sites selected, two were excluded: one refused (see below) and the other was unable to participate in the study because of flooding.

### Study procedures

For each community selected, approval of the chief was first sought. All residents were then sent a letter from the Fiji Ministry of Health describing the purpose and methods of the study. Eight Fijian senior nurses were selected and trained on the diagnosis and treatment of scabies and impetigo, as well as on research methodology, and divided into four survey teams, one per geographical division. Each team, made of two nurses and one village health worker, visited the study site and provided an overview of the project and invited residents to participate. The study coordinator attended several visits to assist the team, assess the quality of the survey procedures and take photos of scabies cases. Those who consented provided demographic details and underwent a complete skin examination by trained personnel. All exposed areas of the skin were examined and genitals and perimamillar regions were only examined if participants described symptomatic itching in these regions and consented for examination. All examinations were conducted in private areas using opaque screens. Participants were also invited to complete a brief clinical questionnaire regarding previous diagnosis and treatment of scabies and other skin conditions, and participants who were illiterate or with low vision were provided assistance to complete the questionnaire by the study staff Consent for children and adolescents under 18 years of age was provided by parents or guardians. All clinical results were made available to the local community care nurses, and study participants diagnosed with scabies or other skin conditions were referred for treatment to the local nursing station.

### Case definitions

Scabies was diagnosed on the basis of characteristic clinical findings, defined as the presence of pruritic inflammatory papules with a typical acral distribution [19]. Impetigo was defined as papular, pustular or ulcerative lesions surrounded by erythema with or without the presence of crusts, frank pus or bullae. Impetigo lesions were counted and classified according to their number (1 to 4, 5–20 or more than 20 lesions) and graded as flat/dry (old, almost healed sores with no crust), crusted (yellow or red scab over a skin sore) or purulent (wet or moist sores with obvious presence of pus). If a mixture of impetigo types were seen, the dominant sore type was recorded.

### Statistical analysis

Participant characteristics were summarised by demographic categories (age, sex, ethnicity, Fiji administrative division, urban/rural) and compared to the distribution of these characteristics in the 2007 national census data. Prevalence of scabies and skin sores were calculated for the whole population and by each of the demographic categories. Univariate and multivariate logistic regression models were used to identify factors independently associated with a high prevalence of scabies and impetigo. The age and division specific prevalences were used to calculate national estimates of scabies and impetigo prevalences, standardising against age and division specific national census figures. The population attributable risk was calculated to estimate the association between scabies and skin sore prevalence [20]. All statistical analyses were conducted using STATA 12.0 (College Station, TX, USA).

### Ethical approvals

Ethical approval for this study was obtained from the Fiji National Research Ethics Review Committee and the Fiji National Health Research Committee and reviewed by the St Vincent’s Hospital (Sydney) Research and Ethics Committee. Persons 18 years and over, willing to sign an informed consent form, and village members under 18 years old with a consent form signed by a parent/guardian were included in the study.

## Results

A total of 75 sites were included in the survey, with a median proportion of 82% of the official resident population examined in each village, with an overall participation rate of 78.2%. In 72 of the villages the proportion examined was 60% or more. A total of 10,887 study participants were enrolled. As shown in Table 1, the demographic characteristics of the study sample were broadly comparable to that of the 2007 census population in regard to age and ethnicity, but there was substantial over-representation of younger age groups (54% of the sample was aged less than 15 years compared to 29% from census data), and of people from the Northern division of Fiji (40.0% of the sample vs. 16.2% from census data).

**Table 1:** Demographic characteristics of the study sample compared to national census data.

Factor			Study (n = 10,887)		Census (n = 837,271)
		**n**	%	%
**Gender**	Female	5491	50.4	49.0
	Male	5396	49.6	51.0
**Ethnicity**	iTaukei	7580	69.6	56.8
	Indo-Fijian	3183	29.2	37.5
	Other	124	1.2	5.7
**Age (years)** *	<5	1023	9.4	9.9
	5–9	2408	22.1	9.3
	10–14	2448	22.5	9.8
	15–24	1587	14.6	19.1
	25–34	1256	11.5	16.4
	35–49	1131	10.4	19.5
	>49	951	8.7	16.0
**Division**	Western	3036	27.9	38.2
	Central	2955	27.2	40.9
	Eastern	538	4.9	4.7
	Northern	4358	40.0	16.2
**Location**	Rural	6304	57.9	50.7
	Urban/Peri Urban	4583	42.1	49.3

*data on 83 participants were not recorded

**Fig 1:**
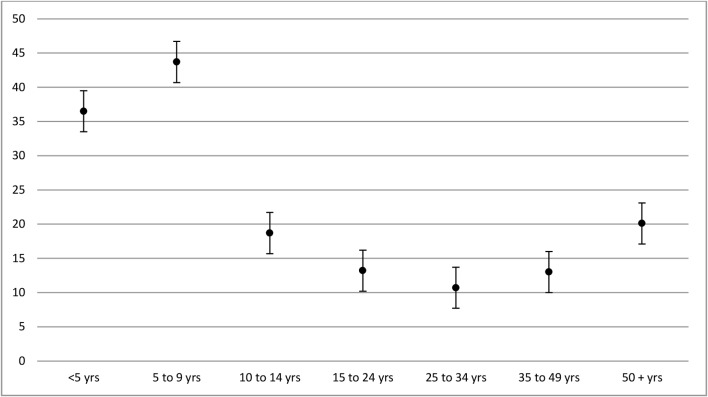
Distribution of scabies by age.

Scabies was observed in 2,564 participants (23.6%) of the population surveyed (Table 2). Prevalence was highest in children aged five to nine years (43.7%, adjusted odds ratio, OR, 3.7 when compared to those aged over 49 years, Fig. 1). Scabies was nearly twice as common in iTaukei Fijians (24.7%, adjusted OR 2.7 when compared to the Indo-Fijian population). Scabies was most prevalent in people living in the Northern division (28.5%), in people living in rural areas (25.6%) and in females (24.8%). Taking into account the census distribution of age and geographical location by direct adjustment, the estimated national prevalence of scabies was 18.5% (95% CI 14.7–22.9). There were no cases of crusted scabies detected in this study.

**Table 2:** Prevalence of scabies in Fiji.

Factor		Sample	Participants with scabies			Adjusted OR† (95% CI)
		**n**	**n**	%	**95% CI**	
Total		10,887	2564	23.6	22.8–24.3	
Gender	Female	5491	1361	24.8	23.6–26.0	1.2 (1.1–1.3)
	Male	5396	1203	22.3	21.2–23.4	1
Ethnicity	iTaukei	7580	2077	27.4	26.4–28.4	2.7 (2.4–3.0)
	Indo-Fijian	3307	487	14.7	13.6–16.0	1
Age (years)*	<5	1023	373	36.5	33.5–39.5	2.5 (2.0–3.0)
	5–9	2408	1053	43.7	41.7–45.7	3.7 (3.0–4.4)
	10–14	2448	457	18.7	17.4–20.6	1.0 (0.8–1.2)
	15–24	1587	209	13.2	11.5–14.9	0.6 (0.5–0.8)
	25–34	1256	134	10.7	9.0–12.5	0.5 (0.4–0.6)
	35–49	1131	147	13.0	11.1–15.1	0.6 (0.5–0.8)
	>49	951	191	20.1	17.6–22.8	1
Division	Western	3036	644	21.2	19.8–22.7	1.0 (0.8–1.3)
	Central	2955	577	19.5	18.1–21.0	1.0 (0.8–1.3)
	Northern	4358	1240	28.5	27.1–29.8	1.3 (1.0–1.7)
	Eastern	538	103	19.1	15.9–22.7	1
Location	Rural	6304	1611	25.6	24.5–26.7	1.2 (1.1–1.4)
	Urban / Peri-urban	4583	953	20.8	19.6–22.0	1

*data on 83 participants were not recorded;

†adjusted odds ratio calculated by gender, ethnicity, age, division and location

The prevalence of impetigo was also high, with lesions observed in 19.6% of participants (Table 3). Impetigo followed a similar age distribution to scabies, being more common in children aged five to nine years (34.2%, adjusted OR 2.2). Similar to scabies, impetigo prevalence was twice as high in the iTaukei Fijians (22.8%, adjusted OR 2.4), in females (20.5%), in people living in rural areas (21.4%) and in the Northern division (23.7%). When taking into account the census distribution of age and geographical location the national prevalence of impetigo was 18.5% (95% CI 12.3–20.2). The majority of impetigo lesions were mild (53.5%) and flat/dry (54.8%), although severe cases were not uncommon (16.2%) and over a quarter of the sores were purulent (26.6%, Table 4).

**Table 3:** Prevalence of impetigo in Fiji.

		Sample	Participants with impetigo			Adjusted OR† (95% CI)
		**n**	**n**	%	**95% CI**	
Total		10,887	2133	19.6	18.9–20.4	
Gender	Female	5491	1127	20.5	19.5–21.6	1.2 (1.1–1.3)
	Male	5396	1006	18.6	17.6–19.7	1
Ethnicity	iTaukei	7579	1730	22.8	21.9–23.8	2.4 (2.2–2.8)
	Indo-Fijian	3308	371	11.2	10.2–12.3	1
Age (years)*	<5	1023	236	23.1	20.5–25.8	1.2 (1.0–1.5)
	5–9	2408	823	34.2	32.3–36.1	2.2 (1.9–2.7)
	10–14	2448	411	16.8	15.3–18.3	0.8 (0.7–1.0)
	15–24	1587	133	8.4	7.0–9.9	0.6 (0.5–0.8)
	25–34	1256	153	9.6	10.4–14.1	0.4 (0.3–0.5)
	35–49	1131	174	15.4	13.3–17.6	0.5 (0.4–0.6)
	>49	951	200	21.0	18.5–23.8	1
Division	Western	3036	553	18.2	16.9–19.6	1.2 (0.9–1.5)
	Central	2955	463	15.7	14.4–17.0	1.1 (0.8–1.4)
	Northern	4358	1035	23.7	22.5–25.0	1.4 (1.1–1.8)
	Eastern	538	82	15.2	12.3–18.6	1
Location	Rural	6304	1351	21.4	20.4–22.5	1.2 (1.1–1.3)
	Urban/Peri-urban	4583	782	17.1	16.0–18.1	1

*data on 83 participants were not recorded;

†adjusted odds ratio calculated by gender, ethnicity, age, division and location).

**Table 4:** Association between scabies and impetigo.

		Total(n = 10,887)		Participants with scabies(n = 2564)*		Participants without scabies(n = 8323)	
		n	%	n	%	n	%
**Impetigo**		2133	19.6	2021	78.8	112	1.3
**Number of lesions**	<5	1142	53.5	1059	52.4	83	74.1
	5–20	646	30.3	622	30.8	24	21.4
	>20	345	16.2	340	16.8	5	4.5
**Lesion type**	Flat/Dry	1170	54.8	1116	55.2	54	48.2
	Crusted	396	18.6	371	18.4	25	22.3
	Purulent	567	26.6	534	26.4	33	29.5

The presence of impetigo was strongly associated with a diagnosis of scabies (relative risk, RR, 58.6, 95% CI 48.7–70.5). The population attributable risk of scabies as a cause of impetigo based on the national survey was 93.1%.

Study participants who reported a previous diagnosis of scabies (n = 2923, 26.9%) were more likely to have scabies infestation at the time of the survey (51.4%) compared to those who did not recall a prior scabies diagnosis (13.3%, RR 3.4, 95% CI 3.2–3.6). Similarly, participants reporting prior treatment (n = 1586, 14.6%) had a higher prevalence of scabies than those who did not (RR 2.7, 95% CI 2.6–2.9). Of those who reported prior treatment, the most common treatment was topical cream of an unspecified type (39.1%) followed by various local plant-based treatments (36.9%) and benzyl benzoate (12.8%). At the time of the survey, permethrin cream was unavailable through the publicly funded clinics and could only be purchased through private pharmacies. Ivermectin, an oral treatment for scabies, is not available in Fiji.

## Discussion

This study is the first national survey of scabies and impetigo prevalence conducted in any country and indeed the only one that we are aware of that is based on a rigorous sampling methodology and covers a substantial population and geographic area. Our survey confirms that scabies and impetigo are widespread problems in Fiji. While we observed that children are the most affected population group, no age group is free of scabies or impetigo, and there is an indication that prevalence increases after middle age. Scabies and impetigo are highly prevalent across all geographical divisions and both genders and in both the main ethnic groups.

High levels of scabies and related bacterial infections have previously been documented in tropical countries with low or medium socio-economic status and in disadvantaged populations, particularly in countries in the Pacific region [21]. However, all previous studies were conducted in smaller, localised areas, such as single provinces, schools or villages [21]. Prior reviews of the prevalence of scabies and impetigo have consistently documented high levels of both diseases in school-aged children [9,21]. Only a limited number of studies have described the tendency for scabies to reappear in older age groups, as we observed [7,14,22]. A possible explanation for this apparent phenomenon is that under the typical family structure in these countries, older community members frequently care for children, and are thereby exposed to a heightened risk of transmission of scabies from children. A further possible explanation is that repeated infestation in childhood leads to protective immunity in adulthood but this wanes in the elderly, although there are very few data to support this hypothesis. Consistent with our findings, previous studies in Fiji have documented that scabies and impetigo are most prevalent in the iTaukei Fijian population [8,14,21]. The reason for this is not clear but may be possibly linked to a higher number of children per family and the tendency to live in single-room houses. Other bacterial infections including pneumonia, invasive streptococcal and staphylococcal infections, and rheumatic heart disease have been observed to be more common in iTaukei Fijians [6,23–25].

Impetigo is common in Fiji. Over 15% of participants in this study had more than 20 impetigo lesions and over one quarter of participants had purulent lesions. This study clearly documents that scabies is the main driver of bacterial skin infection in Fiji with scabies contributing 93% of the risk of impetigo. This striking finding has considerable implications for public health efforts to control impetigo; that is, successful control measures directed at scabies would likely translate into significant reductions in the burden of impetigo.

The survey had a number of methodological limitations. Our sample had an over-representation of younger children and an under-representation of older people, possibly due to sampling of areas with primary or secondary schools in proximity of the selected sites, and the relative absence of adults due to regional work commitments. Further, there may have been weaknesses in the diagnosis of scabies and impetigo. Although diagnosis was entirely clinical, and did not use a microscope or scraping of scabies mites, we employed senior nurses, who received intensive training in relevant methods, and were paired to conduct the examination. However no further validation was conducted and we did not conduct any bacteriology tests to confirm the presence of bacterial infection of impetigo lesions.

The Fiji Ministry of Health received a preliminary report of this survey Romani [10], and has since undertaken a number of measures to increase community and health care worker awareness and knowledge of scabies. The Government’s Integrated Management of Childhood Illness guidelines now include an assessment and treatment guide for skin health with a focus on scabies and bacterial skin infection [19]. Education about scabies has been conducted through the hospitals, clinics and health centres.

This large, nationwide survey of scabies and impetigo provides comprehensive data on the prevalence of these diseases in Fiji indicating that one-fifth of the population is affected at any one time, and that nearly 50% of school-aged children are affected. This tremendous burden of disease at a population level strongly supports the need for investment into research to investigate the best strategies for public health control of scabies in communities where resources are limited and scabies and it complications endemic. Our data suggest that an effective public health control measure for scabies will also likely lead to a considerable reduction in the burden of impetigo.

## Supporting Information

S1 Checklist STROBE Checklist. (PDF)
